# KETOS: Clinical decision support and machine learning as a service – A training and deployment platform based on Docker, OMOP-CDM, and FHIR Web Services

**DOI:** 10.1371/journal.pone.0223010

**Published:** 2019-10-03

**Authors:** Julian Gruendner, Thorsten Schwachhofer, Phillip Sippl, Nicolas Wolf, Marcel Erpenbeck, Christian Gulden, Lorenz A. Kapsner, Jakob Zierk, Sebastian Mate, Michael Stürzl, Roland Croner, Hans-Ulrich Prokosch, Dennis Toddenroth

**Affiliations:** 1 Chair of Medical Informatics, Friedrich-Alexander-University Erlangen-Nürnberg (FAU), Erlangen, Germany; 2 Medical Centre for Information and Communication Technology, Universitätsklinikum Erlangen, Erlangen, Germany; 3 Department of Pediatrics and Adolescent Medicine, UniversitätsklinikumErlangen, Erlangen, Germany; 4 Department of Surgery, Division of Molecular and Experimental Surgery, Friedrich-Alexander-University Erlangen-Nürnberg (FAU), Erlangen, Germany; 5 Department of General, Visceral, Vascular and Graft Surgery, University Hospital, Magdeburg, Germany; University of Pennsylvania, UNITED STATES

## Abstract

**Background and objective:**

To take full advantage of decision support, machine learning, and patient-level prediction models, it is important that models are not only created, but also deployed in a clinical setting. The KETOS platform demonstrated in this work implements a tool for researchers allowing them to perform statistical analyses and deploy resulting models in a secure environment.

**Methods:**

The proposed system uses Docker virtualization to provide researchers with reproducible data analysis and development environments, accessible via Jupyter Notebook, to perform statistical analysis and develop, train and deploy models based on standardized input data. The platform is built in a modular fashion and interfaces with web services using the Health Level 7 (HL7) Fast Healthcare Interoperability Resources (FHIR) standard to access patient data. In our prototypical implementation we use an OMOP common data model (OMOP-CDM) database. The architecture supports the entire research lifecycle from creating a data analysis environment, retrieving data, and training to final deployment in a hospital setting.

**Results:**

We evaluated the platform by establishing and deploying an analysis and end user application for hemoglobin reference intervals within the University Hospital Erlangen. To demonstrate the potential of the system to deploy arbitrary models, we loaded a colorectal cancer dataset into an OMOP database and built machine learning models to predict patient outcomes and made them available via a web service. We demonstrated both the integration with FHIR as well as an example end user application. Finally, we integrated the platform with the open source DataSHIELD architecture to allow for distributed privacy preserving data analysis and training across networks of hospitals.

**Conclusion:**

The KETOS platform takes a novel approach to data analysis, training and deploying decision support models in a hospital or healthcare setting. It does so in a secure and privacy-preserving manner, combining the flexibility of Docker virtualization with the advantages of standardized vocabularies, a widely applied database schema (OMOP-CDM), and a standardized way to exchange medical data (FHIR).

## Introduction

The tremendous increase in computing power and parallelization of processes in the last decades have led to an increase in clinical decision support models, patient-level predictions and other machine learning applications. Their widespread utilization also increased the diversity. Computations now can range from simple statistics and reference interval calculations to complex neural networks. However, less time has been spent on the integration in clinical environments directly at the point of care. Typically, deployment of such statistical computations has faced several political, social, economic and organizational challenges [1–3].

A number of attempts have been made to design deployment infrastructures. Soto et al. [4] developed the EPOCH and ePRISM platforms, allowing researchers to train and deploy risk adjustments, which are translated directly into web-based decision support and reporting tools. ePRISM focuses on general regression and does not provide an application programmable interface (API) to integrate with existing electronic health record (EHR) data. Velickovski et al. [5] developed a clinical decision support system for patients suffering from chronic obstructive pulmonary disease. It uses a service-oriented architecture for early detection and diagnosis of this particular disease. More recently, Baldow et al. [6] developed MAGPIE, a platform allowing researchers to develop Unix command line executable computations, which are embedded into Systems Biology Markup Language (SBML). MAGPIE focuses on the development of models, which have to be supplied manually with parameters when executed. Gibson et al. [7] constructed NiftyNet, a TensorFlow-based platform that allows researchers to develop and distribute deep learning solutions for medical imaging. Khalilia et al. [8] used a service-oriented architecture based on OMOP on FHIR [9] to design an infrastructure for training and deployment of pre-determined specific algorithms. Their use of modern technologies like FHIR [10], which provides a REST interface and OMOP [11] as a common data standard, allows them to train algorithms on standardized data. The approach taken by Khalilia et al. focuses on providing a set of standardized machine learning approaches, which are built automatically using the given input features.

In summary, the architectures above focus on a particular disease or type of algorithm, use proprietary data formats, or provide customized application programming interfaces (API) tailored to one specific use case. A general development environment, which integrates well with standardized data provisioning, for researchers to implement new algorithms and perform a broad spectrum of statistical analysis is still needed.

More recently, the German Medical Informatics Initiative (MI-I) [12] has begun funding multiple consortia to develop infrastructure for integrating clinical data from patient care with research data not only within a hospital, but also across hospitals and research institutions [13–15]. The fundamental component defined within the four funded MI-I consortia to tackle this challenge are data integration centers (DIC). They shall establish the technical and organizational infrastructure at each participating hospital providing multiple services. These include data integration, data harmonization, standardized data repositories, consent management, and ID management. To make use of the wealth of clinical, imaging, and molecular data, some of the four consortia have defined use cases in which working groups of the consortia will apply novel machine learning and other statistical approaches to generate patient subgroup classifications or predictive/prognostic tools for dedicated patient cohorts. The work presented in this paper was conducted within the MIRACUM consortium, because one of its goals is the deployment of such new statistical methods directly in clinical settings as an innovative new IT solution integrated in a hospital’s EHR system.

At the Friedrich-Alexander University Erlangen-Nürnberg (one of the MIRACUM partners) we therefore designed and implemented a generic platform to develop and deploy arbitrary computations, aiming to respond to the heterogeneity of statistical analysis. The platform has been named KETOS after the Greek word for sea monster (as it relies on Docker (whale) to implement lightweight encapsulated virtual development environments for researchers). KETOS aims to provide a flexible environment to perform statistical analysis, develop clinical decision support models based on structured data and make them available in a clinical care context.

The objective of this paper is to illustrate the design features of KETOS and its implementation. As we will demonstrate with three example evaluations, the flexibility of the proposed architecture supports a researchers need to quickly respond to new model building and deployment demands as the research infrastructure grows, and new algorithms, and statistics are developed.

## Methods

In order to make machine learning and decision support models deployable and patient data accessible to researchers, the focus should be on providing the same input data format for the training and the calling process. The environment for this needs to be secure and interoperable. Due to the heterogeneity of the model building environments, it is essential to provide a framework that allows any researcher to invent their own algorithms and enables them to concentrate their efforts on research rather than on technical deployment details.

**Fig 1:**
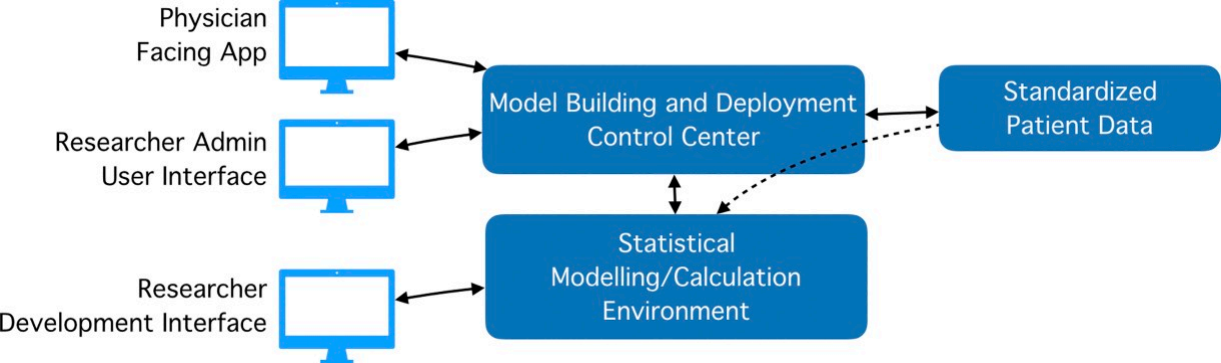
Abstract research and deployment platform.

In order to provide such a research and deployment platform (shown in Fig 1), the following requirements need to be satisfied:

- *Standardized Patient Data*: Standardized input data for development and deployment
- *Statistical Modelling/Calculation Environment*: A mechanism to create and save models for later use
- *Model Building and Deployment Control Center*: A central webservice to manage computations and data access
- *Researcher Admin User Interface*: A user interface for our central webservice for administration
- *Researcher Development Interface*: An environment that allows the researcher to develop and deploy models
- *Physician facing App*: Applications, which use the webservice to access our models in a secure manner and to share them with physicians.

**Fig 2:**
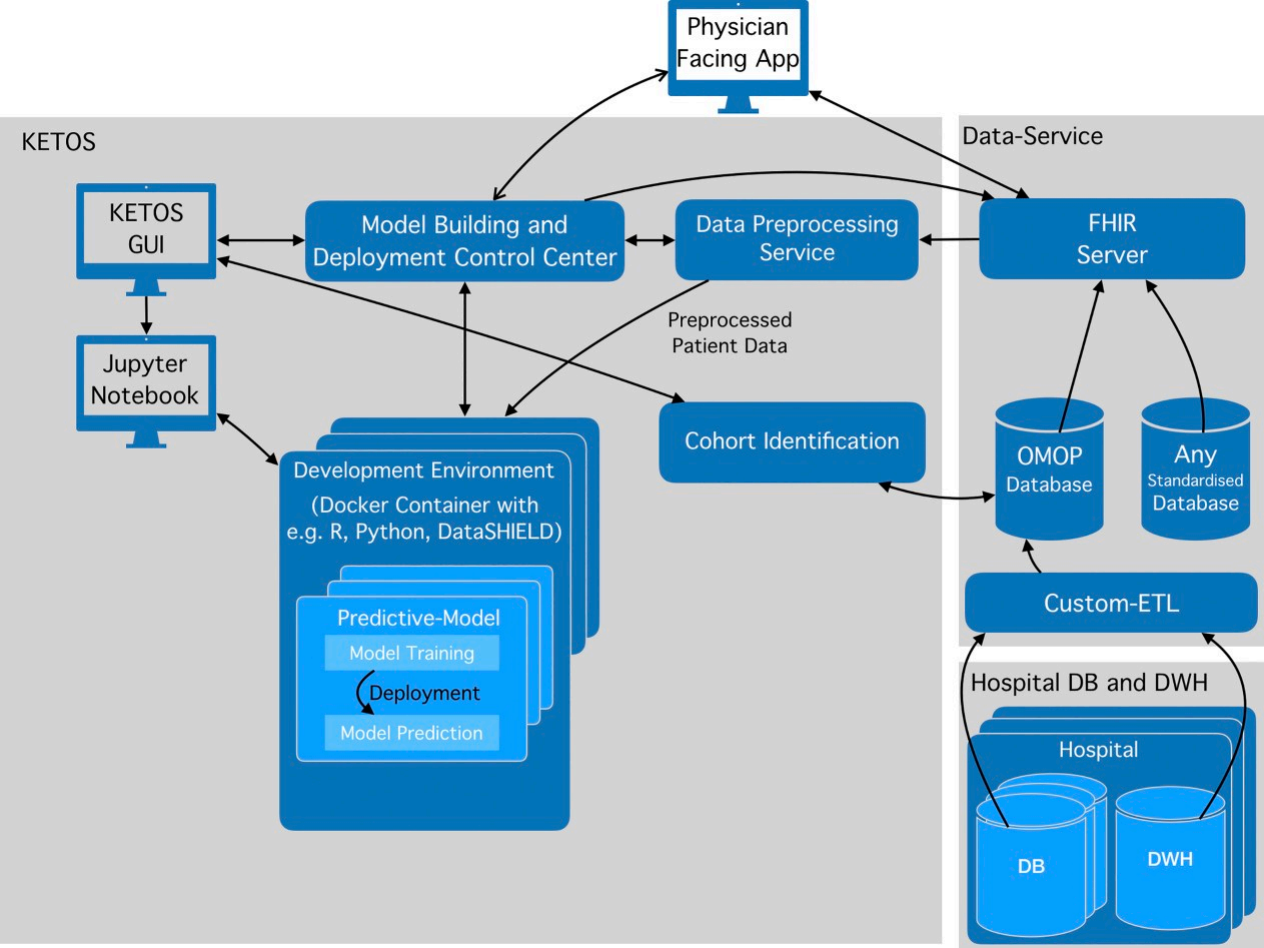
KETOS architecture.

The feasibility of this approach has been demonstrated by implementing a prototype using the architecture illustrated in Fig 2. To provide patient data in a standardized manner we used the OMOP common data model, which has been chosen by the OHDSI project [16], the European EHDEN project [17], and the MIRACUM consortium [18]. This data source is augmented with the widely used FHIR web service standard for patient data to make data available to researchers and end-user applications. We developed a *model building and deployment control center* web service, and a *user interface*, which allows researchers to create their own preconfigured virtual development environments via Docker containers. The researchers are given access to the *development environments* (e.g. R, Python) for statistical data analysis, training and deployment via *Jupyter Notebook* (interactive cell-based code development in a web browser) [19]. A data *preprocessing service* extracts the required data from the *OMOP database* via the *FHIR* webservice and converts it into an analysis-friendly column-based format.

The architecture we propose in Fig 2 aims to close the gap between clinical research and the application of decision support and machine learning models, whilst providing a secure research infrastructure to scientists. It allows statisticians, biometricians and collaborating computer scientists to develop and train their statistical models on pre-processed, standardized data and to deploy them via a web service within a hospital’s IT infrastructure. The KETOS graphical user interface allows researchers to create a new development environment consisting of base tools like R, Python and DataSHIELD. The access to the environments via Jupyter Notebook allows researchers to individually install additional packages. They can then request data from a preprocessing service and adjust the pre-processed data further as needed by specifying respective functions inside their Jupyter Notebook. These alterations to the environment are saved and can be retrieved at a later time. Thus, KETOS supports the management of multiple modelling environments by multiple users. Once a model is built, a researcher can specify a calling function, which can be invoked by the physician facing app for seamless integration (deployment process). This allows the researchers to process any data in the same way regardless of whether they are creating, training or executing the model. The development of reliable decision support models requires the input data to be of high quality and to have the same structure across the training and deployment process. Opposed to this, hospital data is typically heterogeneous [20]. KETOS aims to respond to this challenge by using a FHIR server and relying on a preprocessing service, that prepares FHIR [10] resources for analysis. The preprocessing service is therefore independent of the underlying data schemas. In our example implementation, we use the GT-FHIR service [9] in combination with the OMOP-CDM [11], which was designed to facilitate research (see Fig 2, Data-Service). OMOP, the source database, uses standardized vocabularies, such as the *Systematized Nomenclature of Medicine* (SNOMED [21]) and *Logical Observation Identifiers Names and Codes* (LOINC [22]). As a result, all computations are based on standardized data, which can be understood and identified clearly by researchers and clinicians alike.

The HL7 FHIR standard, which KETOS connects to via the GT-FHIR service, has recently been developed to address shortcomings of the previously developed HL7 clinical care document standard. It aims to improve interoperability, and its lightweight nature and direct use of common data formats, like JSON and XML, let it easily integrate with lightweight webservices. This underlines FHIR’s suitability for web based platforms, such as KETOS, and will allow models built to easily integrate with web and mobile application. Saripalle concludes that the FHIR standard can close the interoperability gap between the many different healthcare entities [23].

### Example use cases and data

To demonstrate the application of the proposed architecture, we applied our prototype to three example models, see results section of this paper. For our first example we generated a synthetic dataset and for our third example we used a publicly available dataset. For our second example we performed our analysis on an anonymized dataset of 300 colorectal cancer patients from the University Hospital Erlangen. This investigation was conducted according to the principles expressed in the Declaration of Helsinki, and the study was approved by the local ethics committee (Ethik-Kommission der Friedrich-Alexander-Universität Erlangen-Nürnberg, - 3914).

## KETOS training and deployment process

**Fig 3:**
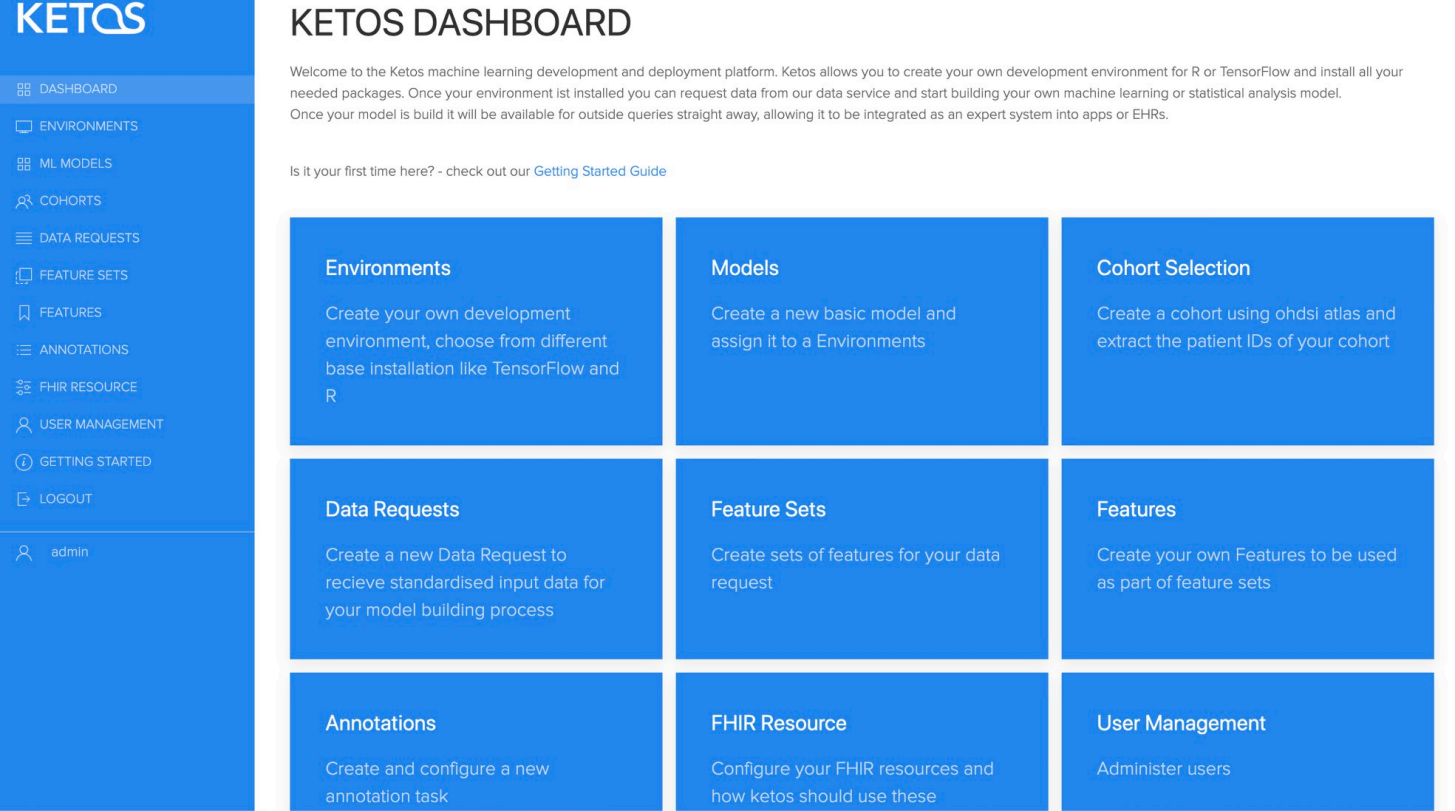
KETOS GUI Dashboard.

The process consists of four main steps, as reflected in the GUI (see Fig 3):

1. Create a development environment (GUI: Environments, Models)
2. Define and retrieve data (GUI: Cohort Selection, Features, Feature-Sets, Data Requests)
3. Adjust data further and train models (GUI: Jupyter Notebook [access via link from Environments)])
4. Deploy and make the computation available via web service (GUI: Models, Environment Jupyter Notebook)

### Step 1 –Create a development environment

**Fig 4:**
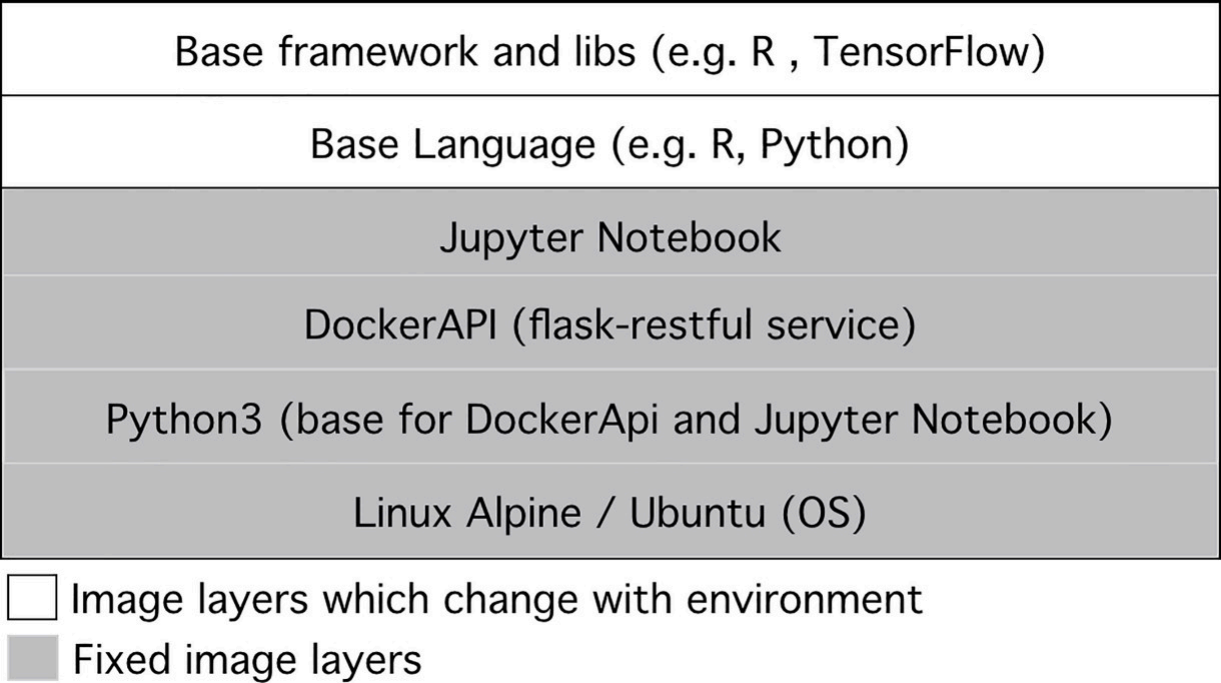
Composition development environment docker image.

Researchers select a new Docker image via the GUI and initiates a new development environment. We currently provide three base image options: TensorFlow, R and DataSHIELD. Each image contains a base Ubuntu/Alpine Linux operating system, as well as a preinstalled Jupyter Notebook. It is augmented with a web service for the communication with the model (see Fig 4). The rest of the environment can be configured directly by installing packages via Jupyter Notebook. Once set up, a new model can be initialized, which adds a new folder and an example implementation ipynb (Jupyter Notebook) file.

### Step 2 –Request data

In the second step, the researchers define a group of patients in the cohort selection process, which is currently supported by the OHDSI ATLAS tool [24]. Features, i.e. patient data variables of interest to the researcher, are then defined in the KETOS GUI. They are combined to form a feature set which can be used to request the data for the patients. A data request is sent via the preprocessing service, which generates a so-called *prepared dataset* by converting the FHIR resources into an analysis-friendly CSV format. The requested data can then be loaded directly into the environment. Alternatively, researchers have the option to upload their own data via the Jupyter Notebook.

### Step 3 –Process data and train model

Once requested, data can be analyzed and modified in the Jupyter Notebook. One example is the reduction of possible outcome values by grouping them together as implemented in [25]. The platform supports researchers in saving and loading their work within the respective environment, the file system of which is mapped to the KETOS host for persistence. This allows KETOS to save and load any model without the platform knowing how storage and retrieval is implemented inside the respective development environment. It further enables the researcher to use KETOS for research and computation only, while not requiring data to leave the respective institutional boundaries, as the whole training process will take place on the KETOS server.

### Step 4 –Deploy model

In the final step of the KETOS process researchers can deploy their computation and make it available as a web service endpoint by labelling it in compliance with the Jupyter Notebook (model.ipynb) file provided in the first step. Once deployed, a call to the central KETOS web service (*Model building and Deployment Control Center*) endpoint will call the prediction function of the environment Docker API. The prediction function will then execute all respectively labeled cells of the Jupyter Notebook and return the output as the prediction result.

## Restricting data access and ensuring patient privacy

With the GDPR [26] taking effect across Europe, preserving a patient’s privacy, while still being able to analyze data, is becoming ever more challenging. KETOS takes an important step forward as it can be completely embedded into a hospital’s IT infrastructure without compromising data privacy. By using modern authentication methods and HTTPS, access to KETOS can be controlled and restricted to registered researchers within the hospital. Furthermore, new data requests can only be generated by an administrator, e.g. the head of a scientific work group. Data requests of a work group can then be reviewed to ensure that the generated data is kept in a standardized format and within the hospital’s IT infrastructure, and that it complies with all the privacy regulations. This allows the respective KETOS host to monitor the data usage more closely within each hospital. Further, data requests as prepared datasets make it easy to archive the data and monitor its use.

## Results

We implemented and deployed the above described system at the University Hospital Erlangen and implemented and evaluated 3 models to demonstrate its use in a clinical setting. Additionally, we shared the examples with adjusted data sets publicly to demonstrate the potential of our platform.

### Example model 1 –hemoglobin reference intervals

**Fig 5:**
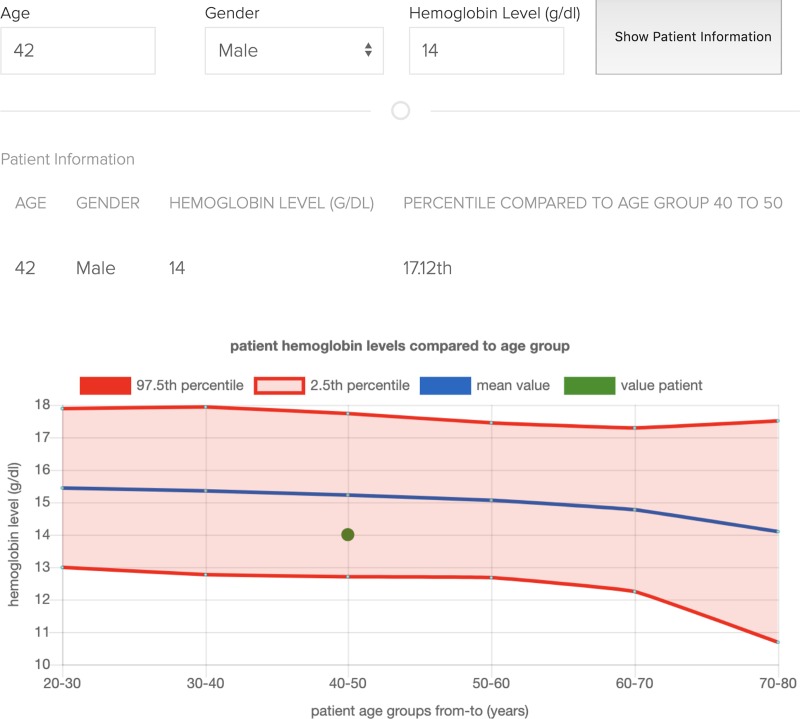
Hemoglobin reference interval application.

We developed a clinical decision support prototype for individual patients’ laboratory test results. The interpretation of numerical laboratory test results requires comparison to reference intervals, i.e. comparison to the 2.5^th^ and 97.5^th^ percentiles of a comparable group of healthy controls. Reference intervals, established using data mining of laboratory information systems, can offer practical, ethical, financial and medical advantages compared to conventional reference interval creation approaches in which healthy persons are recruited for blood sampling [27]. Here, we used KETOS and the *Reference Limit Estimator* developed by the *German Society for Clinical Chemistry and Laboratory Medicine* (DGKL) [28] to establish age- and gender-specific hemoglobin reference limits from local hospital data, and deployed them within Erlangen University Hospital. The deployed prototype features a web based password-protected hemoglobin percentile calculator (only accessible from within the hospital network), which compares the patient data inserted in the app with the local reference intervals provided to the app via the KETOS-built reference calculations, and allows physicians to compare their patients’ test results with a matching age and gender group (see Fig 5). This is a prototype and a fully featured version would include reference intervals for other laboratory analytes. We were able to integrate and deploy the algorithm provided by [28] within hours in a secure hospital environment. A demonstration version of this web application prototype (https://ketos.ai/hb/home) and the deployed model can be accessed via the KETOS website (https://ketos.ai/environments - example_1_hb_ref_intervals). The example data was synthetically created to resemble a similar distribution to patients at the Erlangen University Hospital, without guarantees of clinical correctness. In contrast to example 2 below, the example data was uploaded with the Jupyter Notebook for a one-time reference calculation. Once calculated the reference intervals were then deployed using the calling function of the model. The calculation of the patient percentile compared to a reference interval then takes place in the front-end application. This further demonstrates the versatility of the platform acting as a model delivery system only, as in this case data is entered in a physician facing app and is not gathered from a patient database.

### Example model 2 –predicting colorectal cancer outcomes

To demonstrate the potential of our platform to easily integrate new predictions with arbitrary datasets, we used a dataset of 300 colorectal cancer patients from the University Hospital Erlangen and transformed it to an OMOP-CDM representation (see Fig 2 –Data-Service). Of these patients, 150 (50%) suffered from rectal carcinomas and 150 (50%) from colon carcinomas. The age ranged from 20 to 97 years, with an average age of 68 years. Of all patients, 100 (33%) had experienced a relapse, and 76 patients (25%) died. The mapped dataset included the following feature groups identified as relevant in one of our previous works [25]: gene expression (n = 59 genes) and cancer type (n = 1: colon/rectum). We mapped the Gene Expression data as measurements in the OMOP-CDM and created a custom vocabulary for the data based on the Human Genome Organization (HUGO) Nomenclature [29]. For the publicly available dataset the real gene names where replaced with indices 1–59 and the other features were mapped to a self-created “KETOS Example” vocabulary. Then we established an environment for R and two example machine learning models, similar to the ones from our previous work [25], to predict outcomes in colorectal cancer patients.

In order to demonstrate how different machine learning outcomes could be predicted, we created one model to predict survival time (in month after diagnosis) and another to predict a relapse event (Yes/No). To build these models we used the dataset described above, which included all 59 genes (gene names changed) and cancer type to predict survival time and relapse (see supplement example_2_relapse.zip and example_2_survival.zip). The respective Jupyter Notebooks can be accessed online via our demo server (https://ketos.ai/environments - example_2_ColRecCancer). To further demonstrate the versatility of the KETOS approach the first model was generated using the *mlr* R library [30] and the second built using the *caret* library [31] (see supplementary information example2_survival.zip and example2_relapse.zip). Both follow the same pattern for building and deployment. First, the data is read from within the modelling environment using the preprocessing service to access the data and adjusted accordingly. The dataset is then randomly split 80/20 into training and testing datasets. Second, multiple algorithms are then trained for the predictive outcome using standard functions of the respective packages. The prediction results are evaluated using specific performance metrics. Finally, the most successful model is chosen and called in the predict function. In our process we found that our models could be ported to the KETOS environment with no need for additional adjustments. The biggest challenge for the performance was the FHIR preprocessing, as the multiple rest calls and merging of the JSON FHIR resources into a standardized table type format took the largest amount of time from any call, as evidenced by the time taken to prepare the analysis dataset for the 300 patients with 65 columns taking several minutes. Despite the slow performance of the data generation via FHIR and the preprocessing of larger datasets, similar to Khalilia et al. we measured prediction response times for a single patient of a few seconds on the commodity hardware (i7 2,6GHz, 16GB Ram) we used. Further, as we synthetically increased the number of features from 100 to 1000 features the execution time grew linearly with the number of features (from 11 to 91 seconds).

### Example model 3 –DataSHIELD: Towards privacy preserving distributed data analysis

**Fig 6:**
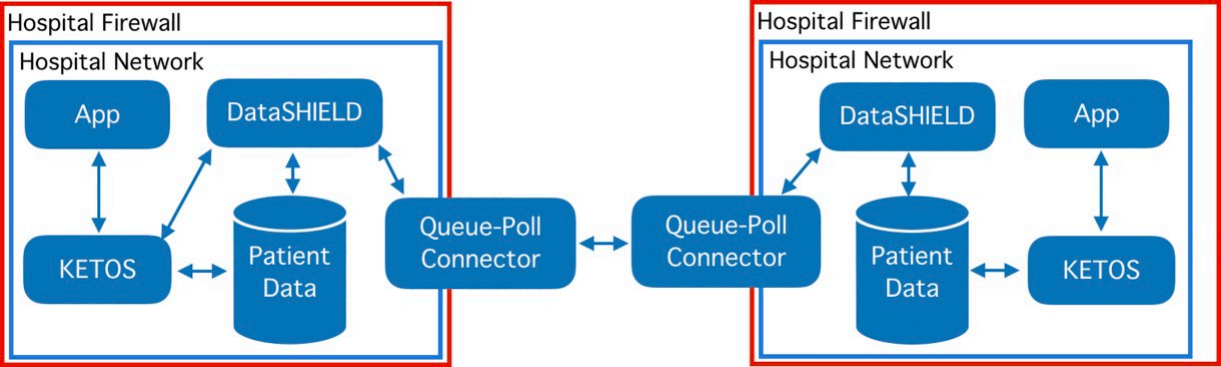
KETOS and DataSHIELD—privacy preserving distributed data analysis and model deployment.

In previous work we have established a DataSHIELD analysis network with multiple German university hospitals of the MIRACUM consortium and combined them to one privacy preserving analysis network [32]. DataSHIELD allows for privacy-preserving distributed analysis by only allowing aggregated statistics to leave the boundaries of an institution. For our third exemplary evaluation we integrated KETOS into such an extended DataSHIELD-based distributed analysis network, by setting up a KETOS environment as the analysis client of the network (Fig 6). This permits the training of decision support models on a federated network with a focus on preserving patient privacy using DataSHIELD and then deploying them locally in a hospital network secured by firewalls, HTTPS and password protection. In detail the computation and deployment process is as follows: First, researchers request data to be loaded into the DataSHIELD network. This would currently still be supported by a manual loading process into the DataSHIELD environments own database (Fig 6 –DataSHIELD). Researchers then initiate a DataSHIELD environment inside KETOS, load the appropriate packages and connect to the DataSHIELD environment from within the Jupyter Notebook of the new KETOS environment. Subsequently, the analysis can then be performed using the DataSHIELD R libraries to connect to other DataSHIELD installations within a consortium via the Queue-Poll Connector. Following analysis a model can be created inside the KETOS environment. Finally, researchers specify a predict function inside the Jupyter Notebook, which loads the saved model, gathers the data using a data request, previously specified in the KETOS model via the GUI, and returns the response. If an authorized application (Fig 6 App) then sends a request via KETOS, the system will gather the patient data from its FHIR server, execute the previously specified prediction function and return the response. To provide an example version to the general public, we have deployed a general linear model (GLM), by connecting the publicly available KETOS instance to our publicly available DataSHIELD data warehouse instance. The resulting GLM was then deployed for individual patient data using the coefficients provided and supplied with data using the FHIR preprocessing service of the KETOS instance. To create this example we have split the Breast Cancer dataset from the R mlbench package [33], which is based on the Wisconsin Breast Cancer Dataset [34], into a training and test dataset and uploaded the training dataset to our DataSHIELD warehouse. We then mapped and uploaded the test dataset to our OMOP database and deployed the model using the KETOS infrastructure. The example version is available here (https://ketos.ai/environments - example_3_DataSHIELD).

## Discussion

We presented KETOS, a platform providing secure access to health research data and allowing researchers to train and deploy statistical models within a clinical research environment.

The platform allows researchers to use tools already part of the data-scientists workflow (Jupyter Notebook, Python and R) to develop custom statistical computations without restrictions. It improves on potential drawbacks of the Khalilia et al. [8] and ePRISM approaches [4], which do not allow researchers to develop their own algorithms, perform research only, and adjust input data freely. KETOS can be tailored to meet specific requirements and does not confine the building and optimization process. This results in a quicker response to new research. While Velickovski et al. [5] focused on chronic obstructive pulmonary disease, KETOS supports arbitrary computations, ranging from prospective and concurrent to retrospective research. Finally, the platform allows researchers to rely on standardized data, which makes models with larger amounts of input parameters possible, as the data can be collected automatically. This would not be supported by for example the MAGPIE [6] platform, where our colorectal cancer example with 65 features would not be feasible, as a clinician would need to input all variables to call a prediction function. Further, it can be used as a method to supply restricted data access to researchers while at the same time making use of a large pool of data without compromising patient privacy.

We have demonstrated the different aspects described above with three example analyses. In the first example, we demonstrated the deployment of an algorithm and the resulting model provided by a third party. This example also showed the integration with a physician facing web application. In our second example, we demonstrated how different commonly used machine learning R packages (mlr and caret) could be used to create an individual machine learning pipeline to analyze gene expression data combined with other patient data. One advantage of this approach was, that some of the adjustments made, e.g. dropping certain columns (like patient ID) from the dataset or loading the necessary libraries could be repeated in the prediction call function. This meant that adjustments needed for training and prediction could easily be integrated into the KETOS process. The flexibility of the infrastructure also allowed us to have different responses for the different prediction outcomes. In the third example we integrated KETOS seamlessly with a larger existing privacy preserving data analysis tool. This was implemented using the R DataSHIELD library, allowing one to combine cross-hospital analysis on aggregated data with secure local deployment using patient level data. Overall it can be concluded that different types of algorithms and models integrate well with the KETOS architecture and that the flexibility of Jupyter Notebook allows researchers to implement their own machine learning or model building pipelines, which can differ considerably from the examples given here.

The platform can also be used to facilitate the workflow of data provisioning, data analysis, and multicenter research, as any KETOS computation will run in any KETOS instance with the same data standardization (i.e. SNOMED CT, LOINC, self-created vocabulary). The proposed platform can therefore play an integral part of bringing analysis to the data as, for example, envisioned by the MIRACUM consortium. It does this by building on the proposed infrastructure of the DICs being established across Germany by the MI-I. While our example database is currently an OMOP schema, KETOS actually connects to FHIR, which makes it independent of the underlying database. While the FHIR format is relatively new and has seen several changes over the last years, the release of version 4.0.0 (R4) has seen the definition of the first normative resource descriptions [35]. This indicates that the standard is now becoming more stable. Three large technology companies: Google, Microsoft and Apple have adopted or incorporated FHIR into their software [36–38]. The Office of the National Coordinator for Health Information Technology (ONC) just published data at the end of last year supporting that FHIR is already widely spread across the United States health system and stating: “About 82 percent of hospitals and 64 percent of clinicians use these developers’ certified products” [39]. In the MIRACUM consortium FHIR will allow us to establish a central data storage from which to fill other research databases like OMOP and i2b2 and improve interoperability between different consortia across the MI-I initiative, as FHIR has been agreed on as the format for inter consortia data exchange [40]. KETOS enables researchers to create statistical models, while preserving patient privacy and adhering to data security regulations, as well as deploy them securely in a clinical setting supporting translational research.

This is the basis for development in one hospital and applying additional training or deployment in another. We have shown that our platform integrates easily with existing distributed privacy preserving data analysis solutions like DataSHIELD [41]. To our knowledge, the herewith described framework is the first of its kind in Germany that allows researchers to train across multiple hospitals in a privacy-preserving manner, without the patient data having to leave the hospitals and then deploy the resulting decision supporting models within a hospital network to which only authorized physicians have access.

### Limitations

Sharing across KETOS instances is only possible insofar as the different instances use the same underlying data schema. However, the current use of OMOP by a large German university hospital consortium [42], as well as the definition of structured FHIR profiles by the MI-I [40] provide a good starting point. The reliance on a standardized format means that any prediction is dependent on an extract transform load (ETL) process, which is heavily influenced by the amount of input data and may take up to days for the data to be processed, thus currently limiting the execution process to non-real-time data. This is something that could be overcome by improving the ETL performance or creating a duplicate smaller subset of all data for real time predictions, which can be analyzed on a more regular basis. The preprocessing of the FHIR resources, as shown in example two is the main bottleneck for performance and should be improved in future research to make working with even larger datasets feasible. Further, as KETOS does not restrict the model building process inside the Jupyter Notebook, the performance of each model will depend strongly on the libraries used by the respective researchers. More specialized datatypes, such as imaging data, would require more complex preprocessing pipelines, like NiftyNet. The platform places no restrictions on the JSON data schema returned as the output. This, while making the platform versatile, also means that researchers cannot expect a predefined structure. Thus, a close collaboration between the person calling the deployed model and the researcher is needed during the development process to clarify the output format. This process can be facilitated in the future by providing more meta information and structured response types.

### Future directions

We aim to extend the KETOS preprocessing service and feature selection process via FHIR (to support e.g. time series data). KETOS provides a user management and access control; however, we are currently also working on connecting the system to a self-hosted SMART app platform [43] and integrating the end user applications with existing clinical information systems. The integration with existing privacy preserving tools, like DataSHIELD, would allow KETOS to become part of a larger research infrastructure, which only permits registered researchers inside the MIRACUM-established DICs to use requested data. The building and deployment capabilities of KETOS should then be tested further. We are planning to recreate and extend the hemoglobin reference interval use case using DataSHIELD once the data is available harmonized across different institutions. The use of FHIR also supports the analysis of data across consortia in the MI-I and allows us to improve the preprocessing service of KETOS in this pursuit. Further, development should focus on scaling the system and providing a high-performance cluster. In the future we would like to make more statistical models available in healthcare routine in a FHIR compatible way. In this pursuit new concepts will have to be developed to write model generated data (e.g. predictions and classifications) back to FHIR, as, for example, the FHIR risk assessment resource does not lend itself well to writing back non-risk related predictions or classifications.

## Conclusion

The KETOS platform provides a novel approach to data analysis, training, and deployment of decision support models in the healthcare sector by combining the advantages of standardized vocabulary (SNOMED, LOINC, self-created vocabulary), a structured database (OMOP-CDM), and a standardized way to deliver medical data (FHIR) with the flexibility of Docker virtualization in one single framework. It implements the basis for distributed, highly scalable data analysis with a major focus on patient privacy as the sensitive data does not have to leave the premises. In this work we presented a prototype, which demonstrates that decision support models can be built in a standardized way and deployed in a secure way, while allowing for maximum flexibility in the research process.

## Supporting information

S1 File example_1_refInt.zip. ketos_metadata/metadata.json (json)
model.ipynb (ipynb)
prog (directory).
(ZIP)

S2 File example_2_relapse.zip. colRec_relapseModels (Rdata)
data_all.csv (csv)
ketos_metadata/metadata.json (json)
model.ipynb (ipynb)
test_data_relapse.csv (csv)
training_data_relapse.csv (csv)
utils.R (R).
(ZIP)

S3 File example_2_survival.zip. colRec_mlrModels (Rdata)
data_all.csv (csv)
ketos_metadata/metadata.json (json)
model.ipynb (ipynb)
test_data.csv (csv)
training_data.csv (csv)
utils.R (R).
(ZIP)

S4 File example_3_ds.zip. ds_ketos_model (Rdata)
ketos_metadata/metadata.json (json)
model.ipynb (ipynb).
(ZIP)

S5 File getting_started.zip. ketos_metadata/metadata.json (json)
ketos_model (Rdata)
model.ipynb (ipynb)
test_data.csv (csv)
training_data.csv (csv).
(ZIP)
