# Correction: KETOS: Clinical decision support and machine learning as a service – A training and deployment platform based on Docker, OMOP-CDM, and FHIR Web Services

**DOI:** 10.1371/journal.pone.0225442

**Published:** 2019-11-13

**Authors:** Julian Gruendner, Thorsten Schwachhofer, Phillip Sippl, Nicolas Wolf, Marcel Erpenbeck, Christian Gulden, Lorenz A. Kapsner, Jakob Zierk, Sebastian Mate, Michael Stürzl, Roland Croner, Hans-Ulrich Prokosch, Dennis Toddenroth

The third author’s name is spelled incorrectly. The correct name is: Philipp Sippl.
